# Pharmacological Role of Functionalized Gold Nanoparticles in Disease Applications

**DOI:** 10.3390/molecules27051551

**Published:** 2022-02-25

**Authors:** Wen-Chin Ko, Su-Jane Wang, Chien-Yu Hsiao, Chen-Ting Hung, Yu-Jou Hsu, Der-Chen Chang, Chi-Feng Hung

**Affiliations:** 1 School of Medicine, Fu Jen Catholic University, New Taipei City 24205, Taiwan; wck@cgh.org.tw (W.-C.K.); med0003@mail.fju.edu.tw (S.-J.W.); 2 Division of Cardiac Electrophysiology, Department of Cardiovascular Center, Cathay General Hospital, Taipei 10630, Taiwan; 3 Department of Nutrition and Health Science, Chang Guang University of Science and Technology, Taoyuan 33303, Taiwan; mozart@gw.cgust.edu.tw; 4 Research Center for Food and Cosmetic Safety and Research Center for Chinese Herbal Medicine, Chang Gung University of Science and Technology, Taoyuan 33303, Taiwan; 5 Graduate Institute and Department of Pharmacology, National Taiwan University College of Medicine, Taipei 10051, Taiwan; oh510230@gmail.com; 6 PhD Program in Pharmaceutical Biotechnology, Fu Jen Catholic University, New Taipei City 24205, Taiwan; s16179263@gmail.com; 7 Department of Mathematics and Statistics and Department of Computer Science, Georgetown University, Washington, DC 20057, USA; chang@georgetown.edu; 8 School of Pharmacy, Kaohsiung Medical University, Kaohsiung 80708, Taiwan

**Keywords:** gold nanoparticles, nanogold, antioxidant, anti-inflammation, anti-angiogenesis, cancer

## Abstract

Gold has always been regarded as a symbol of nobility, and its shiny golden appearance has always attracted the attention of many people. Gold has good ductility, molecular recognition properties, and good biocompatibility. At present, gold is being used in many fields. When gold particles are as small as several nanometers, their physical and chemical properties vary with their size in nanometers. The surface area of a nano-sized gold surface has a special effect. Therefore, gold nanoparticles can, directly and indirectly, give rise to different biological activities. For example, if the surface of the gold is sulfided. Various substances have a strong chemical reactivity and are easy to combine with sulfhydryl groups; hence, nanogold is often used in biomedical testing, disease diagnosis, and gene detection. Nanogold is easy to bind to proteins, such as antibodies, enzymes, or cytokines. In fact, scientists use nanogold to bind special antibodies, as a tool for targeting cancer cells. Gold nanoparticles are also directly cytotoxic to cancer cells. For diseases caused by inflammation and oxidative damage, gold nanoparticles also have antioxidant and anti-inflammatory effects. Based on these unique properties, gold nanoparticles have become the most widely studied metal nanomaterials. Many recent studies have further demonstrated that gold nanoparticles are beneficial for humans, due to their functional pharmacological properties in a variety of diseases. The content of this review will be the application of gold nanoparticles in treating or diagnosing pressing diseases, such as cancers, retinopathy, neurological diseases, skin disorders, bowel diseases, bone cartilage disorders, cardiovascular diseases, infections, and metabolic syndrome. Gold nanoparticles have shown very obvious therapeutic and application potential.

## 1. Introduction

Particles with a size between 1 and 1000 nm are defined as nanoparticles. There are many substances or organisms belonging to the nanometer level in nature. For instance, the length of a typical carbon–carbon bond ranges from 0.12 to 0.15 nm; the DNA double-helix has a diameter of approximately 2 nm; the smallest cellular life form, *Mycoplasma bacterium*, is around 200 nm in length [1]. Nanoparticles have good in vivo stability and cell uptake efficiency, and are widely used for drug delivery. Metal/metal oxide nanoparticles can also simulate the activity of antioxidant enzymes and catalyze the degradation of superoxide anions and hydrogen peroxide [2,3,4]. The degradation of metal/metal oxide nanoparticles releases metal ions and inhibits inflammation [4].

**Figure 1:**
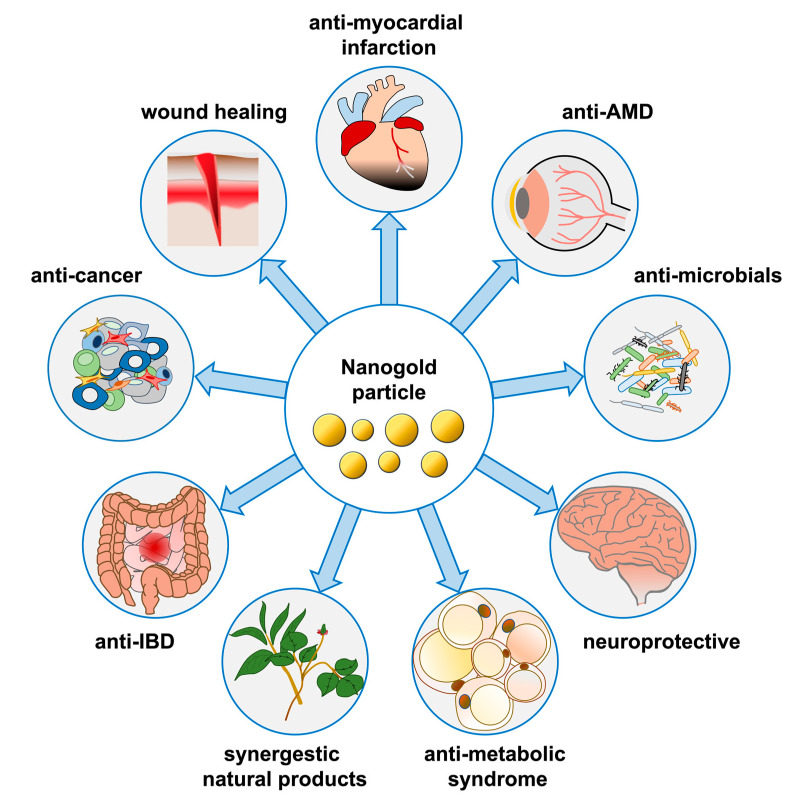
Physiological and pharmacological functions of nanogold in various organ systems and diseases.

Of all the nanoparticles (NP) used in experimental studies, gold nanoparticles (GNPs or AuNPs) seem to be the most effective, with little systemic toxicity. Nanogold-related research articles were published in large numbers around 2000 [5]. Gold nanomaterials include gold nanoparticles, nanoclusters (AuNCs), nanocages, nanorods (AuNRs), nanostar, nanoshells, and nanoplates. Their controllable geometry, optics, and surface chemistry make them very promising for biomedical applications. [6]. Gold at the nanoscale level also exhibits special optical properties. With the difference in nanoscale, gold changes from red to purple. Gold nanoparticles, due to their excellent properties, such as easy synthesis, controllable size, specific surface plasmon resonance, and excellent biocompatibility, have shown fascinating feasibility for the treatment of various diseases [7]. Therefore, the content of this review article is based on the foundational research of nanogold particles in recent years, with the goal of understanding their physiological and pharmacological functions in various organ systems and diseases (Figure 1 and Table 1). This review mainly focuses on the development potential of nanogold in organ diseases of the human body. It is expected that more scholars will conduct further in-depth research in the future, so that the application of nanogold is integrated in treatment options for these organ diseases.

## 2. Retinopathy

The retina is the innermost layer of the eye, spanning the back two-thirds of the eye, and contains the protective internal and external blood retinal barrier (BRB). Nanomaterials can be designed to have properties and characteristics that enable them to pass through the BRB, when delivered by local installation or targeted intraocular injection [8]. Therefore, the application of nano-grade particles in ophthalmology has also been paid great attention in recent years. The two main causes of moderate to severe visual impairment worldwide are retinal diseases, including age-related macular degeneration (AMD) and diabetic retinopathy (DR) [8]. The standard treatment for retinal neovascularization is intravitreal injection of laser and anti-vascular endothelial growth factor (VEGF) preparations. These treatments effectively slow down or prevent the formation of new blood vessels. However, the laser can damage the surrounding retinal tissue and cause persistent discomfort. Frequently required repeated intravitreal injections increase the risk of endophthalmitis and retinal detachment [9]. The eye consists of an anterior segment and a posterior segment. The barriers encountered by the application of therapeutic substances to the retina include corneal and conjunctival epithelium, blood-aqueous barrier (BAB), and blood-retinal barriers (BRB) [8]. BRB is mainly divided into two parts: inner BRB and outer BRB. Nanomaterials can be designed to have properties and characteristics that enable them to pass through the BRB when delivered by local installation or targeted intraocular injection [10]. Gold nanoparticles have proven to have anti-angiogenesis and anti-inflammatory properties and can be used to treat retinal diseases [8]. In the previous laser-induced choroidal neovascularization (CNV) animal model, intravenous injection of gold nanoparticles showed significant anti-angiogenic properties [11]. AuNPs are an effective inhibitor of VEGF-induced RF/6A cell migration through the Akt/eNOS pathway [12]. In C57BL/6 mice, the CNV formed by laser destruction of Bruch’s membrane was reduced after gold nanoparticle treatment. Gold nanoparticles reduced the tube formation and proliferation of human umbilical vein endothelial cells (HUVEC) induced by vascular endothelial growth factor, but did not show RPE cytotoxicity at therapeutic doses. AuNPs inhibited the phosphorylation of ERK1/2, Akt, and FAK in HUVECs [13]. Optical coherence tomography (OCT)-assisted CNV volume measurement at all time points showed a significant reduction in lesion size in the group that received an intravenous injection of gold nanoparticles when compared with controls [11,14]. Most notably, metal nanoparticles with diameters of 20 and 80 nm (0.0065 µg/mL 20 nm AuNPs, 0.4 µg/mL 80 nm AuNPs, 0.0035 µg/mL 20 nm AgNPs, and 0.22 µg/mL 80 nm AgNPs) are toxic to photoreceptor cells in vitro [15]. 

## 3. Neuroprotective Effects: Alzheimer’s Disease and Parkinson’s Disease

The blood–brain barrier (BBB) is semi-permeable, which allows certain substances to penetrate, but prevents certain substances from passing. In the brain, endothelial cells are tightly bound together, and substances cannot penetrate outside the blood vessels. Brain diseases are very difficult to treat. The reason is that the BBB makes it difficult for drugs to be delivered to the brain. Many studies have shown that nanoparticles can smoothly pass through the blood–brain barrier [16]. Therefore, the application of nanoparticles in central nervous system diseases has great potential, and this can be investigated in future studies. Dementia can be classified as degenerative, vascular, and reversible, depending on its cause. Degenerative dementia includes Alzheimer’s disease, frontotemporal lobe dementia, and Lewy body dementia. Alzheimer’s disease (AD) is the most common type of dementia. The underlying mechanism explaining the neurotoxicity induced by β-Amyloid peptide (Aβ) aggregates may be oxidative stress [17,18]. Therefore, the inhibition of Aβ fibril formation and the decomposition of Aβ aggregates are considered an important treatment strategy for AD. Gold nanoparticles have been reported by many researchers as having the function of penetrating through the BBB. In the past, many studies have proven that the physical properties of nanogold (AuNPs) have inhibited the aggregation of Aβ peptides and the degradation of Aβ aggregates [16,18,19,20,21,22]. AuNPs also showed inhibition of acetylcholinesterase and butyrylcholinesterase, contributing to an anti-Alzheimer’s disease effect [23]. In addition, Aβ may induce mitochondrial dysfunction by increasing oxidative stress, so it may be related to neurotoxicity in AD [24]. Oxidative stress is one of the main pathological events, because it contributes to neuronal cell death in AD. Recent studies have found that AuNPs (3–5 nm, 10 ppm) increase the viability of neural stem cells exposed to Aβ, which is related to the decrease in the expression of inflammatory cytokines such as Tumor Necrosis Factor-α (TNF-α) and Interleukin-1β (IL-1β). In addition, AuNPs reduce the Aβ-mediated increase in nuclear factor kappa-B (NF-κB; p65). AuNPs treatment significantly restored inducible nitric oxide synthase (iNOS) and cyclooxygenase-2 (COX-2) levels in human neural stem cells (hNSCs) treated with Aβ. The hNSCs treated with AuNPs were significantly protected from Aβ-induced oxidative stress. In addition, the hNSCs co-treated with AuNPs were significantly protected from the Aβ-induced reduction in the expression of nuclear factor erythrocyte 2 related factor 2 (Nrf2) and the downstream antioxidant target genes (SOD-1, SOD-2, Gpx1, GSH) of Nrf2. In addition, AuNPs reduced the expression of HSP27 and HSP70 genes. In conclusion, AuNPs can reverse the inflammation and oxidative stress induced in hNSCs exposed to Aβ [25]. Parkinson’s disease is one of the most common neuromotor disorders affecting the elderly and the second most common neurodegenerative disease worldwide. GNPs (5–10 nm, 250 μg/mL) were transfected into cells by endocytosis and inhibited apoptosis in PC12 cells and dopaminergic neurons [16]. Gold nanoparticles (100 nm, 5–20 μg/mL) biosynthesized from the rhizome of *Paeonia moutan* potentially inhibited the inflammation in vitro murine microglial BV2 [26]. Gold nanoparticles alleviated the neuroinflammation and improved motor coordination in Parkinson-induced mice [16,26].

## 4. Skin Disorders

Wound healing involves a complicated pathophysiological process that includes inflammation, proliferation, and remodeling. Bacterial infection caused by the lack of proper treatment of acute wounds is one of the main reasons for the formation of chronic wounds. As bacteria can cause inflammation, killing bacteria is an important aspect of initial wound treatment. Quantized gold (QG) can act as an endotoxin antagonist in reducing inflammation and preventing a chronic wound from (re)occurring, after which the wound bed will transition from the inflammation phase to the proliferation phase [27]. Such a gold nanocomposite could be further designed as dual function quantized gold (QG), to bind with LPSs, not influencing the catalytic function of the inner core. The catalytic decomposition of H_2_O_2_ into water and oxygen on small-sized nanogold is more efficient than such decomposition on large-sized catalysts [28]. Wound repair after hemostasis may be complicated by infection; therefore, platelet-like particles (PLP) can be combined with antibacterial gold to develop nanogold composites (NGC) to expand wound healing. These NGC PLPs mimic the morphology of platelets, produce clot shrinkage, show certain antibacterial potential, and are promising materials for preventing post-traumatic blood loss and infection [29]. In the past, we also found that the combination of gold nanoparticles and epigallocatechin gallate (EGCG) and α-lipoic acid (ALA) significantly accelerated the healing of skin wounds in mice. Our findings provide a theoretical basis for the future development of AuNPs and other antioxidant mixtures in the topical treatment of skin wounds [30]. The pathophysiology of acne vulgaris depends on active sebaceous glands, which means that selective destruction of sebaceous glands may be an effective treatment. Microparticles with a 120-nm diameter silica core and a 15-nm thick gold shell were chosen, as they provide strong light absorption at ~800 nm. Microscopy showed preferential thermal damage to sebaceous glands and glands following exposure to 10–50 J cm^−2^, 30 ms, 800 nm diode laser pulses [31]. Therefore, the use of gold nanoparticles has a promising future in acne treatment.

## 5. Inflammatory Bowel Diseases (IBD)

A new gold (III) complex was designed and screened in in vitro studies, using a mouse macrophage cell line, RAW264.7, as well as in vivo, in a dextran sulfate sodium (DSS)-induced mouse model of colitis [32,33]. In lipopolysaccharide (LPS)-stimulated RAW264.7 cells, the complex showed a potent anti-inflammatory profile, as evidenced in neutral red uptake and Griess tests. In the dextran sodium sulfate (DSS)-induced mouse model of colitis, the gold (III) complex (1.68 and 16.8 μg/kg) produced a significant anti-inflammatory effect. The underlying mechanisms could be related to the anti-oxidant effect (as evident by decreasing tissue MDA) and anti-inflammatory potential of AuNPs [33]. Moreover, the gold (III) complex induced changes in the tight junction complex expression in the intestinal wall. This is the first study proving that gold (III) complexes may have therapeutic potential in the treatment of IBD [32]. On the other hand, oral gold nanoparticles (5 nm, 25 μg/kg) can prevent colitis by attenuating the inflammatory response mediated by Toll-like receptor 4 and reactive oxygen/nitrogen substances, but may lead to imbalance of the intestinal flora of mice [34]. Therefore, the above research results show that gold nanoparticles have great potential as a novel therapeutic strategy for the treatment of inflammatory bowel disease.

## 6. Bone Cartilage Disorder

As early as 1929, J. Forestier discovered gold salt as a drug for treating rheumatoid arthritis [35]. Many studies later proved that gold drugs are effective for patients with rheumatoid arthritis. Studies have shown that AuNP (15 nm, 25 μg/kg) can improve the production of inflammatory mediators and oxidative stress in collagen-induced arthritis (CIA) rats [36]. The exact cause of rheumatoid arthritis (RA) is unknown, but inflammatory mediators such as TNF-α, IL-1β, COX-2, and nitric oxide (NO) are responsible for the destruction of articular cartilage. CIA rats were treated with AuNPs, and it was found that the levels of inflammatory mediators, such as TNF-α, IL-1β, COX-2, and the activated transcription factor NF-κB were significantly reduced. In addition, Au clusters (5 mg/kg) can also effectively reduce the inflammatory symptoms of CIA in rats and prevent joint damage, without any obvious side effects [37,38]. On the other hand, nanogold (13 nm, 10 μg) could inhibit the mechanism of RA by binding to VEGF and inhibiting the proliferation and migration of endothelial cells. AuNPs (13 or 50 nm; 3.76 μg) can also inhibit ROS and prevent the destruction of RA synovitis [7]. Therefore, AuNPs can inhibit angiogenic activity, inhibit inflammation, or act as antioxidants, to protect cartilage tissue in arthritis. Studies have shown that AuNPs have the ability to inhibit osteoclasts and are one of the most effective nanoparticles for the treatment of bone tissue diseases [7,39].

## 7. Cancer

The oncology application of nanoparticles, especially gold nanoparticles, has become an important therapy for cancer diagnosis and treatment.

### 7.1. Radiosensitization, Photothermal, and Photodynamic Therapy 

Recent results have shown that the combination of curcumin and glucose nanogold particles (20 μL per 1 mL medium) has great potential for alleviating tumor hypoxia and improving the radiosensitivity of breast cancer stem-like cells, providing an opportunity for the development of new high-efficiency and low-toxicity radiosensitizers [40]. Gold nanoparticles with outer membrane vesicles from E. coli (Au-OMV) (at concentrations of 200 μg mL^−1^ and 2 μg mL^−1^ for Au and OMV, respectively) combined with radiotherapy produce radiosensitization and immunomodulatory effects, and successfully inhibit tumor growth in both subcutaneous GL261 glioma cells tumor-bearing and in situ (brain) tumor-bearing C57BL/6 mice. In GL261 glioma cells, Au-OMV combined with radiotherapy greatly increases intracellular reactive oxygen species (ROS) [41]. Recent experimental results have shown that chitosan-capped gold nanoparticle-loaded doxorubicin (CS-GNPs-DOX, 25 nm; 0.1 mM Au concentration) can enhance the effect of radiotherapy and chemotherapy by increasing DNA double-strand breaks and inducing cell necrosis, to significantly reduce the viability of cancer cells, even at very low radiation doses (0.5-Gray, Gy). In particular, the developed multifunctional CS-GNPs-DOX provides a synergistic solution for cancer treatment, which can effectively deliver doxorubicin to tumor cells and enhance radiosensitizing activity, thereby reducing the dose required for conventional radiotherapy [42]. Binding of GNPs to antitumor agents can prevent their deterioration on their way to tumors, while reducing the formulation’s toxicity to healthy tissues. Photosensitizers (PS) are combined with GNPs (2–4 nm in diameter) of various forms and sizes to synthesize new formulations of PDT. Gold nanoparticles can be a photosensitive drug carrier. The surface of NPs can be made covalent (through linkers containing thiol or amino groups) or non-covalent (such as electrostatic interactions) by various molecules. Active nanoparticles can effectively absorb light energy and increase PS excitation, promoting singlet production of oxygen and free radicals [43]. Gold nanoparticles (about 2–50 nm) have the advantages of reduced non-specific distribution, near-infrared (NIR) activation, and use in multi-faceted cancer photothermal therapy (PTT) and drug delivery systems, which are suitable for PTT of cancer. GNPs also have unique optical properties, such as surface plasmon resonance induction, which results in a significant increase in light absorption/scattering and possibly heating of the NPs. The surface of cancer cells is often covered with a layer of growth hormone receptor protein (such as epidermal growth factor receptor, EGFR). Normal cells rarely express these proteins. An antibody that can recognize EGFR is attached to the surface of nanogold. In humans, this complex can find cancer cells and adhere to the surface of the cancer cells, to form images for use in imaging medicine [44].

### 7.2. Drug Delivery

Gold nanospheres labeled with peptides containing isoAsp-Gly-Arg (isoDGR) are an αvβ3 integrin-binding motif, proving an effective carrier for delivering pro-inflammatory cytokines to the tumor vasculature. In vivo studies performed in a murine model of fibrosarcoma showed that low doses of bifunctional nanoparticles bearing isoDGR and TNF (corresponding to few nanoparticles per cell) delayed tumor growth and increased the efficacy of doxorubicin, without worsening its toxicity. Similar effects were obtained using trifunctional nanoparticles loaded with isoDGR, TNF, and IL12. Mechanistic studies showed that nanoparticles bearing isoDGR and TNF could increase doxorubicin penetration in tumors a few hours after injection and caused vascular damage at later time points [45]. In order to enhance the efficacy of anti-cancer activity, 15-nm spherical gold nanoparticles were used to deliver gallic acid (GA) to cancer cells. Compared with unmodified GA, the ability of GNPs–GA complex to inhibit the growth of cervical cancer cells is reduced. Interestingly, high concentration (150 μM) GNPs-GA is not toxic to normal cells, while GA alone is cytotoxic. In short, GNPs-GA is not as effective as GA in inhibiting the proliferation of cervical cancer cells, but it is not cytotoxic to normal cells. Therefore, gold nanoparticles may be used as an alternative phytochemical delivery agent for cancer treatment, to reduce the side effects of radiotherapy and chemotherapy [46,47]. However, nano-gold loaded with resveratrol (Res-GNPs, 39 nm) has better anti-tumor effects than resveratrol in vitro and in vivo, which may be because gold nanoparticles carry more resveratrol into cells and are located in mitochondria. These results indicate that Res-GNPs have significantly better anticancer effects than Res alone in vitro and in vivo, and may be helpful for the clinical treatment of liver cancer [48]. Pegylated gold nanoparticles (PEGAuNPs, 24 nm, 9.8 nM) combined with chemotherapy drugs (doxorubicin or varlitinib) also have a very good cytotoxic effect on pancreatic cancer cells [49]. Toxin from Naja naja venom (NN-32, IC50: 5.0 µg/mL) combined with gold nanoparticles (18 nm) increased the percentage of cytotoxic activity and apoptosis of two human breast cancer cell lines (MCF-7 and MDA-MB-231) [50].

### 7.3. Modulation of Apoptosis, Angiogenesis, and Migration

The main targets for inhibiting angiogenesis are a group of growth factors, including fibroblast growth factor (FGF), platelet-derived growth factor (PDGF), and most importantly, vascular endothelial growth factor (VEGF), a well-known angiogenesis activator. It is expressed during tumor growth and metastasis. Gold nanoparticles (5–20 nm, 5 nM) were able to inhibit the function of pro-angiogenic heparin-binding growth factors (HB-GFs), such as vascular endothelial growth factor 165 (VEGF165) and basic fibroblast growth factor (bFGF), etc. [51]. The mechanism is that the combination of heparin-binding growth factors (HB-GFs) and gold nanoparticles inhibits its function, due to changes in protein structure [51]. Gold-AbVF (antibody to VEGF) (4 nm, 200 μg/mL) induces significantly higher apoptosis compared to B chronic lymphocytic leukemia (CLL) cells exposed to AbVF or GNP alone. Gold-AbVF-treated cells showed marked downregulation of anti-apoptotic proteins and exhibited PARP cleavage. Gold-AbVF-treated and GNP-treated cells showed nanoparticle internalization in early and late endosomes and multivesicular bodies. Single uncoated gold nanoparticles can induce some degree of apoptosis in CLL cells [52].

AuNPs of 3–5 nm (2–10 ppm) may be potential antitumor agents because of their selective cytotoxicity to melanoma cells and inhibition of cell migration. Furthermore, the migration and motility of melanoma cells induced by platelet-derived growth factor BB were reduced after pretreatment with 3–5 nm AuNPs. Adhesion assays showed that 3–5 nm AuNPs could significantly inhibit the adhesion of melanoma cells to collagen. Thus, 3–5 nm AuNPs may be potential antitumor agents, due to their selective cytotoxicity to melanoma cells and inhibition of their motility [53]. 

On the other hand, gold nanoparticles from green synthesis were assessed for their anticancer activity in a 7,12-dimethylbenz(a)anthracene (DMBA)-induced breast cancer mouse model. *Curcuma longa* was used as an aqueous reducing extract and stabilizer at room temperature, and chloroauric acid (HAuCl4) was used to form AuNP. The results of the study revealed that most AuNPs prepared were spherical, with variations in size (in the range of 10–30 nm). The group treated with AuNPs after breast cancer induction showed degenerative tumor lesions in the breast and lymph nodes [54].

## 8. Cardiac and Vascular Injury

AuNPs in human umbilical vein endothelial cells (ECs) and aortic Ecs inhibited the expression of cell adhesion molecules (CAMs) induced by TNF-α. AuNPs reduced TNF-α-induced intracellular ROS production and the NF-κB signaling pathway, and enhanced CAM protein degradation by increasing their ubiquitination. However, they did not interfere with the mTOR pathway of protein synthesis and the binding of TNF-α to Ecs. This study proved that AuNPs have anti-inflammatory biological activity against vascular Ecs in vitro, and can reduce arterial neointimal hyperplasia during vascular injury in vivo. The serum gold concentration was 99.5 ± 18 ng/mL after three-day oral administration [55]. Moreover, 20-nm citrate-covered gold nanoparticles (cit-AuNP) reduced the adhesion of white blood cells and platelets to cerebral blood vessels, prevented BBB failure, and reduced the concentration of TNFF-α in the brain, as well as the expression of ICAM-1 in circulating polymorphonuclear (PMN) leukocytes and cerebral blood vessels in septic mice [56]. The combination of nanogold and antibiotics may be a potential drug candidate for the treatment of sepsis, to avoid sepsis-related encephalopathy. It was found that 20 nm Au-NPs (800–2400 μg mL^−1^) can protect primary cortical neurons from oxygen-glucose deprivation/reperfusion (OGD/R) damage, possibly by reducing apoptosis and oxidative stress, while activating Akt signaling and mitochondrial pathways. The results of this study indicate that Au-NPs may be a potential therapeutic agent for ischemic stroke [57].

Cyanobacteria extract gold nanoparticle solution has antioxidant and anti-myocardial infarction activities. Rats were injected with isoproterenol to induce myocardial infarction; then they were given cyanobacteria extract or gold nanoparticles. The results showed that gold nanoparticles alone (41.7 nm, 200 mg/kg/day, IP (intra peritoneal)) or in combination with cyanobacteria extract can inhibit the changes in serum cardiac injury markers, electrocardiogram, arterial pressure index, and cardiac antioxidant capacity induced by isoproterenol [58]. On the other hand, CT vascular imaging of coronary arteries using AuNPs that were conjugated to collagen-binding adhesion protein 35 (CNA35) were used for targeting collagen I in myocardial infarction. AuNP signal was still detectable in blood, 6 h after intravenous (i.v.) administration, significantly higher than the half-life of iodine-based drugs (5–10 min). The use of AuNPs as contrast agents has shown potential in imaging systems [59].

## 9. Synergistic Nature Product Activity

Poor bioavailability, easy oxidation, first-pass metabolism, and rapid efflux have always been the main obstacles to natural products in drug development. (–)-Epigallocatechin-3-gallate (EGCG) was attached to gold nanoparticles (25 μM: 2.5 or 1 ppm) (EGCG-npNG), which had a synergistic anti-cancer activity and inhibited the growth of melanoma and bladder cancer tumors, by promoting apoptosis in mouse models [60,61]. For in vivo anti-cancer efficacy in Ehrlich ascites cancer-bearing mice, gold nanoparticles (13.6 nm) can be used as an effective delivery system of EGCG and have good potential to enhance anti-cancer efficacy [62]. EGCG-GNPs (30 nm) showed better anti-osteoclast effects than free EGCG in vitro and in vivo [63].

## 10. Antimicrobials

The small size of AuNPs easily crosses bacterial cell membranes, disrupting their physiological functions and inducing cell death. The exact antibacterial mechanism of AuNPs has not been fully elucidated. However, possible mechanisms include inducing microbial death through membrane damage, generating ROS and oxidative stress, causing cellular organ dysfunction, and changes in gene expression and cell signaling [59,64]. Gold nanoparticles were used in external preparations and did not agglomerate after being added to a cream mixture. Since the electromotive force on the surface of the gold nanoparticles was greater, they remained stable even if they were introduced into the cream mixture. Microbiological tests show that the studied creams containing gold nanoparticles had different bactericidal properties against Aspergillus niger and Saccharomyces cerevisiae. For samples with a concentration of 110–200 mg/kg, the permeability of metal nanoparticles through the dermal membrane was confirmed [65]. As a non-antibiotic technology, contact-based antibacterial drugs can use non-specific interactions with bacterial cells to exert antibacterial activity. This is a forward-looking solution to the global bacterial resistance problem. A very simplified method that has been devised is to consider the direct bonding of cationic guanidine-containing amino acids to the surface of a nano-gold carrier. This design takes into account the direct binding of cationic guanidine-containing amino acids to the surface of the nano-gold carrier. Due to the high density of cationic surface charge, the structure has antibacterial activity [66]. The study combined gold nanoparticles and designed DNA molecular probes to detect specific DNA fragments of Mycobacterium tuberculosis. When a specific DNA fragment of Mycobacterium tuberculosis is present, the gold nanoparticle reagent (13 nm, 0.5 nM) will change its color from red to blue, due to the surface plasmon resonance effect. Therefore, a rapid diagnosis of Mycobacterium tuberculosis is carried out using gold nanoparticles and DNA molecular probes [67].

## 11. Metabolic Syndrome and Others

Studies have shown that novel gold nanoparticles (green-synthesized from Erythrina japonica extract) can reduce blood glucose, liver enzyme concentrations, and white blood cell counts in diabetic rats. In addition, they can significantly inhibit the expression of TNF-α and IL-6 genes in visceral adipose tissue of type 2 diabetes. The results show that Au NPs (9 nm, 2.5–5 mg/kg) are novel and efficient nanomaterials for diabetes management [68]. Gold nanoparticles (21 nm) also possess anti-inflammatory effects by downregulating tumor necrosis factor (TNF) α. A study employed C57BL/6 mice fed a pellet high-fat diet (HFD, 43% as fat) and that were treated daily with AuNPs (low (HFD-LAu) or high (HFD-HAu) dose) via intraperitoneal injection for 9 weeks. The HFD-LAu group showed an 8% reduction in body weight, ameliorated hyperlipidemia, and normal glucose tolerance; while the HFD-HAu group had a 5% reduction in body weight with significant improvements in their glucose intolerance and hyperlipidemia [69]. Nanogold combined with hyaluronic acid and adipocyte targeting peptide can be used for lipolysis. Targeted site irradiation with near-infrared laser selectively induces white fat lipolysis and reduces body weight in obese mice [70]. Nanogold-coated polyethylene glycol injected into adipose tissue can achieve increased effects during liposuction [71]. Recently, the most important applications of nanogold have been in the development of coronavirus vaccines and the detection of viruses. These studies stress the indispensable role that nanogold particles play in biomedicine [59].

## 12. Conclusions

The distribution of nanogold in systemic administration may depend on the particle size. Larger gold nanoparticles may stay in the liver. Small gold nanoparticles will reach various organs throughout the body, including the brain, heart, lungs, spleen, and kidneys; where the smaller particles are further eliminated through the kidneys. Of course, if the nanoparticle reaches various organs and tissues, it can escape the phagocytosis of tissue macrophages. It will further reach the location of tissue disease [59]. Topical applications of nanoparticles, such as ophthalmic formulations, can directly achieve the therapeutic goals discussed in this paper due to their direct contact with the target site. The toxicity of gold nanoparticles may be closely related to factors such as particle size, shape, surface potential, dose, and synthesis method [72]. Therefore, the clinical application of gold nanoparticles requires more detailed toxicological tests to prove its safety. At present, most of the related preparations of gold nanoparticles are still in the clinical trial stage. Most of the nanogold-related drugs approved for clinical use by the FDA are nanogold combined with anticancer drugs (such as paclitaxel) or related proteins used in cancer treatment and diagnosis [73]. Currently, clinical trials and basic research on nanogold are in full swing. In the future, more nano-related preparations will enter the clinical treatment and diagnosis of diseases, so that nanogold and nanogold will play a more prominent role in biomedicine.

## Figures and Tables

**Table 1:** Summary of the effects and mechanisms of gold nanoparticles in diseases.

Diseases	Applications (Future) or Possible Action Mechanisms
Cancers pancreatic, breast, prostate, colon, melanoma, sarcoma, and lung cancers, etc.	Anti-cancer activity; cancer diagnosis; imaging applications; photothermal and photodynamic therapies; anti-cancer drug and gene delivery
Retinopathy age-related macular degeneration (AMD); diabetic retinopathy (DR)	Anti-angiogenesis; anti-inflammation; reduced the VEGF activation and induced cell proliferation and migration
Neurological diseases Alzheimer’s disease; Parkinson’s disease	Inhibited the aggregation of Aβ peptides and the degradation of Aβ aggregates; inhibition of acetylcholinesterase and butyrylcholinesterase; anti-inflammation
Skin disorders	Wound healing; acne; synergistic effect with natural products
Bowel diseases	Against inflammatory bowel diseases (IBD); alleviates the lipopolysaccharide-induced intestinal epithelial barrier dysfunction
Bone cartilage disorders	Rheumatoid arthritis treatment; Promotion and regulation of the differentiation; protection for bone and cartilage tissue; the inhibition of osteoclast; inhibit angiogenic activities, suppress inflammation or serve as antioxidant
Cardiovascular diseases	CT imaging as CT contrast agents; anti-inflammatory biological activity; reduce arterial neointimal hyperplasia
Infections	Antimicrobial effects; overcome microbial drug resistance; detect specific DNA fragments of *Mycobacterium tuberculosis*; antiviral activity, coronavirus vaccines and the detection
Metabolic syndrome	Type 2 diabetes and obesity treatment; improvement in glucose intolerance and hyperlipidemia; lipolysis; more effects during liposuction
